# Risk factors for *Clostridium difficile* infections – an overview of the evidence base and challenges in data synthesis

**DOI:** 10.7189/jogh.07.010417

**Published:** 2017-06

**Authors:** Paul Eze, Evelyn Balsells, Moe H Kyaw, Harish Nair

**Affiliations:** 1 Centre for Global Health Research, Usher Institute of Population Health Sciences and Informatics, University of Edinburgh, Edinburgh, Scotland, UK; 2 Sanofi Pasteur, Swiftwater, Pennsylvania, USA; * Joint first authorship

## Abstract

**Background:**

Recognition of a broad spectrum of disease and development of *Clostridium difficile* infection (CDI) and recurrent CDI (rCDI) in populations previously considered to be at low risk has renewed attention on differences in the risk profile of patients. In the absence of primary prevention for CDI and limited treatment options, it is important to achieve a deep understanding of the multiple factors that influence the risk of developing CDI and rCDI.

**Methods:**

We conducted a review of systematic reviews and meta–analyses on risk factors for CDI and rCDI published between 1990 and October 2016.

**Results:**

22 systematic reviews assessing risk factors for CDI (n = 19) and rCDI (n = 6) were included. Meta–analyses were conducted in 17 of the systematic reviews. Over 40 risk factors have been associated with CDI and rCDI and can be classified into three categories: pharmacological risk factors, host–related risk factors, and clinical characteristics or interventions. Most systematic reviews and meta–analyses have focused on antibiotic use (n = 8 for CDI, 3 for rCDI), proton pump inhibitors (n = 8 for CDI, 4 for rCDI), and histamine 2 receptor antagonists (n = 4 for CDI) and chronic kidney disease (n = 4 for rCDI). However, other risk factors have been assessed. We discuss the state of the evidence, methods, and challenges for data synthesis.

**Conclusion:**

Several studies, synthesized in different systematic review, provide valuable insights into the role of different risk factors for CDI. Meta–analytic evidence of association has been reported for factors such as antibiotics, gastric acid suppressants, non–selective NSAID, and some co–morbidities. However, despite statistical significance, issues of high heterogeneity, bias and confounding remain to be addressed effectively to improve overall risk estimates. Large, prospective primary studies on risk factors for CDI with standardised case definitions and stratified analyses are required to develop more accurate and robust estimates of risk effects that can inform targeted–CDI clinical management procedures, prevention, and research.

*Clostridium difficile* infection (CDI) is considered an urgent public health threat [1]. In 2011, a total of 453 000 incident CDI cases were estimated in the USA alone, with an additional 83 000 first recurrences (rCDI) and 29 300 deaths [2]. In Europe, where rates of CDI among inpatients range from 0.7 to 28.7 per 10 000 patient bed–days, at least 40 000 CDI cases are thought to be missed every year through lack of clinical suspicion and inadequate laboratory testing [3]. Endemic CDI and outbreaks have been reported from all world regions, including Asia, Western Pacific, Latin America, and Africa [4–13]. By increasing the length of hospital stay, in addition to the extra costs of diagnosis, treatment, and in some cases surgery or fatal outcomes, CDI places a large economic burden on health care finances and patients [14]. It is estimated that the total annual hospital management of CDI infection in the US alone is US$ 6.3 billion [15].

Recognition of a broad spectrum of disease and development of CDI in populations previously considered to be at low risk has renewed attention on differences in the risk profile of patients. A substantial proportion of CDI cases, between 20 to 30%, are now considered to be community–associated [16] and at least 25% of incident CDI cases will suffer at least a relapse or first recurrence (rCDI) within 30 days of treatment [17]. In the absence of primary prevention for CDI and limited treatment options, it is important to achieve a deep understanding of the multiple factors that influence the risk of developing CDI and rCDI. Commonly reported risk factors include advanced age, co–morbidities, use of antibiotics, proton pump inhibitors (PPIs), histamine–2 receptor antagonists (H2RA) and exposure to health care settings. Other risk factors have also implicated include obesity [18–20], non–steroidal anti–inflammatory drugs (NSAID) [21,22], vitamin D [23], and the role of host genetics in acquiring CDI [24]. We examined systematic reviews and meta–analyses on risk factors for CDI to provide an overview of the state of the evidence and discuss some of the challenges for epidemiological data synthesis for CDI and rCDI.

## METHODS

We searched the following databases: MEDLINE and EMBASE (Ovid); CINAHL; Cochrane database; and Global Health Library. Our eligibility criteria are detailed in **Box 1** and search terms used for each database are available in Table S1 in **Online Supplementary Document(Online Supplementary Document).**

Box 1 **Inclusion and exclusion criteria**

**Inclusion:**

- Systematic reviews with or without meta–analysis examining risk factors for CDI or recurrent episodes in all age groups
- Published between 1990 to October 2016
- Published in English

**Exclusion:**

- Systematic reviews and meta–analyses that focused on mortality, health care costs or treatment, or colonization by *C. difficile*
- Narrative reviews or those with methods not clearly described
- Randomised controlled trials (RCTs); Observational studies (cohort and case control studies); laboratory experiments and in–vitro studies

## RESULTS

We found that multiple systematic reviews had assessed the same risk factors. Furthermore, meta–analyses provided different estimates of association for similar factors. Thus, we focus our review on the conclusions of the publications rather than numerical risk estimates. **Table 1** provides a summary of the risk factors identified through our review, classified into three main groups. All, but one, of the primary studies in these systematic reviews were conducted in industrialised countries of North America, Europe, and Western Pacific region.

**Table 1:** Examples of systematic reviews and meta–analyses on risk factors for *Clostridium difficile* infection (CDI)

Risk factor	CDI	Recurrent CDI
	**Number of systematic reviews [ref]**	
**Pharmacological risk factors:**		
Any use of antibiotics (broad and specific)	8 [21,25-31]	3 [32-34]
Any use of proton pump inhibitors	8 [21,25-31]	4 [32,33,35,36]
Any use of histamine 2 receptor antagonists	4 [21,36-38]	
Anti–ulcer medications (not specific)	2 [25,37]	1 [34]
Non–steroidal anti–inflammatory drug	2 [21,22]	
Aspirin	1 [21]	
Corticosteroids	1 [21]	
Use of opiate during the last episode of CDI		1 [32]
**Host–related risk factors:**		
Age: ≥65 years	2 [21,31]	3 [32-34]
Age: additional year or decade	1 [25]	2 [32,33]
Chronic kidney disease	2 [21,39]	4 [32-34,39]
Diabetes mellitus	1 [21]	1 [32]
Lymphoma or leukaemia	1 [21,31]	
Solid cancer or malignancy	1 [21,31]	
Severity of co–morbidity	1 [25]	
Inflammatory bowel disease	1 [21]	
Congestive heart disease	1 [21]	
Chronic obstructive pulmonary disease	1 [21]	
Peptic ulcer	1 [21]	
Diverticular disease	1 [21]	
Gastroesophageal reflux disease	1 [21]	
Chronic obstructive pulmonary disease	1 [21]	
Low mean concentration of 25 hydroxyvitamin D	1 [23]	
Female sex		1 [34]
Previous diagnosis of CDI		1 [32]
Additional points Charlson scale		1 [32]
**Clinical interventions or characteristics:**		
Duration of hospitalization	1 [25]	1 [32]
Nasogastric tube feeding	2 [25,31]	1 [33]
Stay in intensive treatment unit	1 [25]	
Non–surgical GI procedure	1 [25]	
Vomiting		1 [32]
Previous GI hospitalization		1 [32]
History of surgery		1 [32]
Leucocytes >20 cells/hpf		1 [32]
High faecal interleukin–8		1 [32]
Previous gastrointestinal procedure		1 [32]
Low day–3 IgM anti–toxin A		1 [32]
Serum albumin <2.5g/dL		1 [32]
Hyponatremia		1 [32]
Lymphopenia		1 [32]
Colonization with vancomycin–resistant enterococci		1 [32]

GI – gastrointestinal, hpf – high power field

### Pharmacological agents

#### Antibiotics

CDI has traditionally been regarded as a complication of antimicrobial therapy, particularly broad–spectrum antibiotics that can disrupt the gut flora in hospitalised patients [40]. Several systematic reviews, with or without meta–analyses, have evaluated the role of different antibiotics by class or generation. The first meta–analysis to quantify the risk, published in 1998 [25], found a strong and statistically significant association between antibiotic use and CDI; the risk of CDI was found to be 6 times higher on average compared to individuals not on antimicrobial therapy. Clindamycin, cephalosporins, and fluoroquinolones were and remain associated with the greatest risk of CDI [21,26–28]. Continued use of CDI high–risk antibiotics during follow–up has also been associated with a statistically significant increase in the risk of rCDI [33]. Consequently, antibiotic stewardship programmes are widely recommended for the prevention of CDI and there is substantial interest in recommendations for which antibiotics should be targeted [41].

As several antibiotics have been associated with CDI and rCDI, it is important to consider a number of issues before targeting specific antibiotics over others as means of prevention. Meta–analyses have found substantial heterogeneity in the studies for most antibiotic classes and generations, limiting the ability to draw conclusions about the risk estimates for specific drugs. Furthermore, there are difficulties in addressing the sources of heterogeneity, as these are wide–ranging: significant pharmacological differences even within generations [41], potential antibiotic selection pressure for particular *C. difficile* strains, such as fluoroquinolones and NAP1/B1/027 [41], increased use of other antimicrobials with unknown effect on CDI [42], and differences in local or national guidelines for antibiotic prescribing. Since much of the literature on risk factors for CDI is based on observational studies, the risk estimates from meta–analyses are both confounded as well as biased.

Interesting findings have emerged from recent meta–analytical approaches estimating the association between antibiotics and CDI in different settings. Differences in strengths of association have been found once data for antibiotics are disaggregated by setting or world region. In the hospital setting, as compared to non–diarrheal controls, clindamycin, cephalosporins, carbapenems, fluoroquinolones and trimethoprim/sulphonamides were associated with at least a 2 times an increased risk of CDI, although confidence intervals for pooled estimates among antibiotic classes overlapped [28]. In the community, these antibiotics were associated with a higher risk for CDI – between 8 to 20 times the risk for clindamycin [21,26,27] and 3 to 5 times for cephalosporins and quinolones, but definition of controls groups varied [21]. Macrolides were associated with a 2– to 3–fold higher and statistically significant risk for community–associated CDI but not for hospital–associated CDI [21,26,27]. Non–significant risks have been reported for tetracycline in either setting [21,26–28]. Aminoglycosides have not been found to be significantly associated with CDI in the health care setting [28], but no evidence were available for community–associated CDI. A greater strength of association between exposure to antibiotics and community–associated CDI in the USA as compared to Europe [21]. Furthermore, stronger associations observed between specific antibiotics and community–associated CDI could be due to less confounding effects inherent to studies within hospitals [43]. Limitations notwithstanding, differing estimates of risk for similar antibiotics for hospital or community–associated CDI underscores the importance of a robust evidence–base for the development of relevant antimicrobial prescribing practices for CDI.

#### Gastric acid suppressors

Acid related upper–gastrointestinal disorders including peptic ulcer disease and gastro–esophageal reflux disease are now mainly treated with PPIs and H2RAs [37,44,45]. Although PPIs are generally thought to have a good safety profile, systematic reviews and meta–analyses suggest otherwise, with an overall significant association between the use of PPIs and CDI [21,35–37,44–47] and rCDI [32,33,35,36]. A statistically significant association between the use of H2RAs and CDI has also been reported in meta–analyses [21,36–38]. Limited evidence on the risk posed by the continuous use of H2RAs on rCDI, or on the relative risk of H2RAs vs PPIs for CDI prevents conclusions from being drawn [36,37]. As for much of the evidence on risk factors for CDI, results are largely from observational studies and, thus, despite the extent of the literature and the plausibility of these findings, confounders such as polypharmacy and comorbidities may still play a role and causality cannot be established. It is important to note that varying strengths of association have been reported in primary studies, reinforcing the need for careful interpretation of meta–estimates. For instance, the association between PPIs and CDI remains significant when stratified by antibiotic use among CDI cases (>80% vs ≤80%) [46] and by study design (case–control studies vs cohort studies) [36,44,46]. However, heterogeneity (defined as >50% or *P* < 0.005) was observed in all subgroup analyses [36,37,44,46], even when unadjusted vs adjusted risk estimates from primary studies were analyzed separately [36]. Similar to antibiotics, regional prescribing practices have the potential to impact meta–estimates. A greater strength of association between exposure to PPIs and community–associated CDI was observed in studies from Europe as compared to the USA.

#### Other drugs

Discrepancies have been reported for the use of NSAIDs as a factor influencing the risk of CDI. The use of aspirin was not significantly associated with CDI in the community but corticosteroids were, as reported in one meta–analysis [21]. However, the use of non–selective NSAID (excluding COX–2 inhibitors) was associated with CDI [22]. Understanding the differences between risk estimates and CDI case definition by setting are extremely important for the accurate assessment of the relevance of study findings.

### Host–related risk factors

#### Age

The well–known role of increased age as a risk factor for incident CDI and rCDI has largely been assessed using two criteria: per additional year or decade, or age ≥65 years [31–34]. The incidence of *C. difficile* hospitalisations among patients ≥65 years increased most sharply among this age group in Finland in the early 2000s [48]. In 2011, rates of CDI were 4–fold higher than among adults aged 45–65 and 13–fold higher as compared to those aged 18–44 in the USA [2]. Furthermore, older age has been associated with an increased risk of CDI with the virulent strain BI/NAP1/027 [31] as well as rCDI [33,34].

Age is a common confounder for which estimates are adjusted in primary studies assessing risk factors for CDI [27,28]. There is great value in having age–stratified estimates of risk, and these have been provided in several meta–analyses. For instance, the risk of CDI in the community associated with antimicrobial exposure was approximately 2–fold higher among older adults (≥65 years of age) as compared with children and younger adults [21]. Furthermore, the risk estimate for the use of NSAIDs was significant based on studies with study populations with mean age of 50 or older, but not significant among those with younger age [22]. Similarly, the risk posed by PPI was more pronounced among adults than in the elderly (≥65 years of age) or children [21]. Analytical approaches to elucidate how the risk of CDI differs among populations are important to better differentiate among populations at risk and the development of targeted recommendations.

#### Comorbidities

The association between CDI and selected comorbidities has also been explored systematically. A systematic review highlighted an increased risk of community–associated CDI cases among patients with inflammatory bowel disease, diabetes, leukemia or lymphoma, renal failure, and solid cancer [21], among a wide range of other comorbidities that have been implicated. Although other study populations are likely to be susceptible, such meta–analytical approaches add to the evidence base by pointing toward specific conditions that may require further attention in the clinical setting and in research studies.

Although there is evidence that lower 25–hydroxyvitamin D (25(OH)D) status increases susceptibility to infectious diseases, the evidence is insufficient to establish an association with CDI. A recent assessment of studies evaluating the role of low 25(OH)D and CDI suggested a significant association with rCDI. However, considering that these were based on three studies only, no differences can be observed between outcomes of CDI in hospital or community settings.

#### Clinical characteristics and interventions

The association between CDI and exposure to health care settings is well recognized. However, the risk posed by different interventions remains poorly understood as the available evidence is potentially confounded by other in–patient associated characteristics. A longer hospital stay is strongly associated with exposure to *C. difficile* spores and the likelihood of colonization [25,49]. However, results regarding the length of stay as a determinant for CDI from primary studies have not been consistent. For instance, patients who developed CDI were hospitalised 2–4 times longer [50], while no differences in hospital stay between CDI patients and non–CDI patients have also been observed [51]. Similarly, one study showed a strong association between CDI and length of hospital stay and ICU stay in univariate analysis but a weak association with a wider 95% confidence interval (CI) was reported in the multivariate analysis [52]. The risk of CDI has been shown to be greater in individuals undergoing invasive procedures, such as abdominal surgery, nasogastric tube placement, mechanical ventilation, all of which are associated with prolonged hospitalization [53,54]. A meta–estimate for the association between CDI or rCDI was not identified for most of the clinical interventions in systematic reviews (**Table 1**). The risk of nasogastric tube feeding and rCDI was estimated in one meta–analysis from data in 3 primary studies and found to be not significantly associated [33].

Despite difficulties in developing a rigid clinical prediction rule for CDI or rCDI, prognostic factors that correlate with poor outcome have been differentiated and informed guidance [55]. Key factors include age, treatment with systemic antibiotics, leucocyte count, albumin, and temperature at time of diagnosis. Furthermore, 5 types of CDI patient groups have been differentiated, based on severity of disease. This classification is important for clinical management and for research.

## DISCUSSION

### Implications of challenges in data synthesis for prevention of CDI and further research

There are two major public health goals in relation to CDI prevention. First, it is important to reduce the total number of cases. Second, it is essential to prevent a poor or fatal outcome of those with severe presentation. The availability of new therapeutic and preventive measures such as immunoglobulin, faecal transplant, and vaccines could help reduce CDI morbidity, mortality and costs. For these strategies to be effective and properly targeted to high–risk patients, evidence on risk factors is necessary.

Varying definitions and reporting levels in primary studies are common issues that challenge or preclude meta–analytical synthesis of the evidence, particularly, the case definition of CDI. In available studies, criteria for the definition can range from hospitalisation records of *C. difficile*, on its own or combined with prescription of oral vancomycin, clinical diarrhea, to laboratory confirmation of CDI, with or without presence of pseudomembranous colitis. An essential first step in the assessment of risk factors for CDI is that future studies adhere to recommended surveillance case definitions for CDI [56] and that efforts are made to support case ascertainment with adequate diagnostic tests [3]. This will not only minimise misclassification bias of asymptomatic patients but also allow for further analytical differentiation of incident CDI and recurrent cases occurring in the community or in the hospital setting. Early detection of severe cases and targeted management, such as early surgical consultation with CDI patients, is essential to prevent poor or fatal outcomes.

CDI data collated by different setting of acquisition have the potential to enable targeted advances in the development of preventive and treatment options. For instance, both probiotics and fidaxomicin have been proposed for the prevention of rCDI among patients at “high risk” of recurrence. Similarly, faecal transplantation methods are also now available for treating rCDI [57]. Better quality data are required to make official recommendations on the use of probiotics [58] and the high cost of fidaxomicin poses restrictions for a cost–effective use if those at high risk cannot be correctly identified [59]. Prevention through vaccination is a promising perspective that could tackle CDI primary infections [60]. More evidence is also required to develop prescribing recommendations of pharmacological agents (with the potential to disrupt the gut flora, such as PPIs and H2RA) in the community that would result in a reduction of the risk of CDI.

Another common limitation in the assessment of risk factors for CDI is that data are from observational studies. Given that this will likely remain unchanged, special attention and consensus on potential confounders and selection of comparator groups are needed in the study design or analysis stages. Evidence–base guidance requires data on risk factors to identify patient characteristics that correlate positively with the severity of disease. Bias is likely to be introduced if study populations are not representative of all potential cases by restricting the pool of CDI cases to those patients with recent antibiotic exposure or controls to those with antibiotic–associated or hospital–acquired, toxin–negative diarrhea.

Age, sex, concomitant medication, and comorbidities are often common confounders adjusted for in primary analyses [33], but data on risk factors for CDI requires further considerations. It is important that parameters on the duration and dosage of drugs associated with a risk of CDI, including antibiotics, PPIs, H2RA, and even NSAIDs, are reported clearly to enable subgroup analyses and their consideration in multivariate analyses in primary studies. Studies and meta–analyses have not been able to account for these factors adequately, thus more research is needed. Features of research that would strengthen the evidence base include matched control groups (particularly by age, gender, and location) as well as an examination of duration and number of drug doses (antibiotics and other drugs). A full assessment of adherence to drug treatments, particularly in the community, is difficult to achieve and its effects minimised as much as possible in both the study design and analysis (eg, using dispensed drugs data rather than prescribed). The importance of a nuanced approach to better understand the role of antibiotics and development of CDI was demonstrated in a recent study, which found a dose–dependent association of cumulative exposure (in terms of doses) and temporal effect (within 6 months prior to diagnosis) of antibiotics use and community–associated CDI [61].

Finally, the vast majority of primary studies aimed at estimating risk factors influencing CDI are conducted in industrialised countries, where prevention is currently focused on judicious antibiotic use. The burden of CDI in developing countries where overuse of antibiotic is also prevalent [62] remains poorly described and the capacity to detect and report its incidence needs is limited [63]. This represents a large knowledge gap in CDI epidemiology. The paucity of evidence on CDI incidence and strength of association of different risk factors is worrisome in view of the potential wide–spread of hyper–virulent strains of *C. difficile* as seen for BI/NAP1/027 [16]. Additional, well–designed studies and standardised surveillance methods [64] that integrate clinical and epidemiological data are required in these settings to assess the role of multiple in–hospital and community factors –which may differ from high–resource settings − that can have an impact on CDI or rCDI.

## CONCLUSION

Several studies provide valuable insights into the role of different risk factors for CDI and meta–analytic evidence of association has been reported for putative factors. However, despite statistical significance, issues of high heterogeneity, bias and confounding remain to be addressed effectively to improve overall risk estimates. Further, given the evolving epidemiology of *C. difficile* world–wide, there is a particular interest in achieving a better understanding of the role of the various factors leading to CDI in the hospital vs the community setting. Thus, there would be great value in large, prospective primary studies on risk factors for CDI with standardised case definitions and stratified analyses to develop more accurate estimates of risk effects that can inform targeted–CDI clinical management procedures, prevention, and research.
