# Supplementary material for: Risk factors for Clostridium difficile infections – an overview of the evidence base and challenges in data synthesis
Source: J Glob Health. 2017 Jun 4;7(1):010417. doi: 10.7189/jogh.07.010417 (PMC5460399; doi:10.7189/jogh.07.010417)
Supplement: Online Supplementary Document [file jogh-07-010417-s001.pdf]

Online Supplementary Document

Eze et al. Risk factors for *Clostridium difficile* infections – an overview of the evidence base and challenges in data synthesis

J Glob Health 2017;7:010417

Table S1: Search strategies by database

**Embase (Ovid Technologies)**	
1. Clostridium difficile/<br>2. Enterocolitis, Pseudomembranous/<br>3. Pseudomembranous colitis.mp.<br>4. Antibiotic associated diarrh*.mp.<br>5. Community acquired clostridium difficile.mp.<br>6. hospital acquired clostridium difficile.mp.<br>7. 1 or 2 or 3 or 4 or 5 or 6<br>8. Risk Factors/<br>9. Risk$.mp.<br>10. infection risk$.mp.<br>11. Risk assessment.mp. or Risk Assessment/<br>12. Odds ratio$.mp. or Odds Ratio/<br>13. 8 or 9 or 10 or 11 or 12<br>14. exp Meta Analysis/<br>15. ((meta adj analy$) or metaanalys$).tw.<br>16. (systematic adj (review$1 or overview$1)).tw.<br>17. 14 or 15 or 16<br>18. cancerlit.ab.	19. cochrane.ab.<br>20. embase.ab.<br>21. (psychlit or psyclit).ab.<br>22. (psychinfo or psycinfo).ab.<br>23. (cinahl or cinhal).ab.<br>24. science citation index.ab.<br>25. bids.ab.<br>26. 18 or 19 or 20 or 21 or 22 or 23 or 24 or 25<br>27. reference lists.ab.<br>28. bibliograph$.ab.<br>29. hand-search$.ab.<br>30. manual search$.ab.<br>31. relevant journals.ab.<br>32. 27 or 28 or 29 or 30 or 31<br>33. data extraction.ab.<br>34. selection criteria.ab.<br>35. 33 or 34<br>36. review.pt.<br>37. 35 and 36<br>38. 17 or 26 or 32 or 37<br>39. 7 and 13 and 38
**Medline (Ovid Technologies, Inc)**	
1. Clostridium difficile/<br>2. Enterocolitis, Pseudomembranous/<br>3. Pseudomembranous colitis.mp.<br>4. Antibiotic associated diarrh*.mp.<br>5. Community acquired clostridium difficile.mp.<br>6. hospital acquired clostridium difficile.mp.<br>7. 1 or 2 or 3 or 4 or 5 or 6<br>8. Risk Factors/<br>9. Risk$.mp.<br>10. infection risk$.mp.<br>11. Risk assessment.mp. or Risk Assessment/<br>12. Odds ratio$.mp. or Odds Ratio/<br>13. 8 or 9 or 10 or 11 or 12<br>14. Meta-Analysis as Topic/<br>15. meta analy$.tw.<br>16. metaanaly$.tw.<br>17. Meta-Analysis/<br>18. (systematic adj (review$1 or overview$1)).tw.<br>19. exp Review Literature as Topic/	20. 14 or 15 or 16 or 17 or 18 or 19<br>21. cochrane.ab.<br>22. embase.ab.<br>23. (psychlit or psyclit).ab.<br>24. (psychinfo or psycinfo).ab.<br>25. (cinahl or cinhal).ab.<br>26. science citation index.ab.<br>27. bids.ab.<br>28. cancerlit.ab.<br>29. 21 or 22 or 23 or 24 or 25 or 26 or 27 or 28<br>30. reference list$.ab.<br>31. bibliograph$.ab.<br>32. hand-search$.ab.<br>33. relevant journals.ab.<br>34. manual search$.ab.<br>35. 30 or 31 or 32 or 33 or 34<br>36. selection criteria.ab.<br>37. data extraction.ab.<br>38. 36 or 37<br>39. 20 or 29 or 35 or 38<br>40. 7 and 13 and 39
**Cumulative Index to Nursing and Allied Health Literature, CINAHL (EBSCO Ltd)**	

1. (MH "Meta Analysis") OR "Meta analysis/"<br>2. (MH "Literature Review")<br>3. (MH "Systematic Review") OR "(systematic adj (review or overview)).tw."<br>4. S1 OR S2 OR S3<br>5. (MH "Risk Factors") OR "Risk factor$"<br>6. "Risk$" OR (MH "Risk Assessment")<br>7. (MH "Odds Ratio")<br>8. (MH "Relative Risk")<br>9. S5 OR S6 OR S7 OR S8<br>10. (MH "Clostridium Difficile") OR "Clostridium difficile/"	11. (MH "Enterocolitis, Pseudomembranous")<br>12. (MH "Enterocolitis")<br>13. (MH "Antibiotics, Combined") OR "Antibiotic associated diarrh*.mp."<br>14. (MH "Community-Acquired Infections") OR "Community acquired clostridium difficile.mp."<br>15. (MH "Cross Infection") OR "hospital acquired clostridium difficile.mp."<br>16. S10 OR S11 OR S12 OR S13 OR S14 OR S15<br>17. S4 AND S9 AND S16
**Cochrane Database**	
(Clostridium difficile AND Risk factors)	
**Global Health Library**	
1. (Clostridium difficile AND Risk factor AND Systematic review)<br>2. (Clostridium difficile AND Risk factor AND Meta-analysis)	
